# Testosterone supplementation improves glucose homeostasis despite increasing hepatic insulin resistance in male mouse model of type 2 diabetes mellitus

**DOI:** 10.1038/nutd.2016.45

**Published:** 2016-12-12

**Authors:** M Pal, S Gupta

**Affiliations:** 1 Molecular Science Laboratory, National Institute of Immunology, New Delhi, India

## Abstract

Clinical studies have revealed that testosterone supplementation had a positive effect on glucose homeostasis in type 2 diabetes mellitus (T2DM), but did not address how testosterone supplementation affected insulin responsiveness in the liver, a key glucose homeostatic organ. In this study, we aimed to study the effect of testosterone supplementation on hepatic insulin responsiveness and glucose homeostasis through liver in male high-fat diet-induced T2DM mice. Testosterone treatment to T2DM animals showed reduced hepatic glucose output. Testosterone inhibited the insulin signaling in liver, thus increased insulin resistance. However, testosterone treatment inactivated GSK3α independent of PI3K/AKT pathway and inhibited FOXO1 By interaction of androgen receptor to FOXO1 and downregulated PEPCK, causing repression of gluconeogenic pathway, which is otherwise upregulated in T2DM, resulted in better glucose homeostasis.

## Introduction

Liver is one of the major organs involved in glucose homeostasis in the body. During extended fasting, the liver converts pyruvate to glucose, by a process called gluconeogenesis, to maintain normoglycemic level where phosphoenolpyruvate carboxykinase (PEPCK) being the rate-limiting enzyme. Under normal conditions, once the normoglycemia is attained, insulin inhibits further hepatic glucose production by inhibiting gluconeogenesis. However, in type 2 diabetes mellitus (T2DM), the body is not able to effectively utilize insulin to maintain normoglycemic level, and the hepatic glucose output is not in the ambit of control of insulin and leads to hyperglycemia, which is reflected by higher fasting blood glucose level (BGL).^1, 2, 3^ Clinical reports have shown that there is an association between testosterone levels and metabolic syndrome in men, and testosterone deficiency leads to T2DM. In these studies, testosterone-deficient men, who also had T2DM, when given androgen replacement therapy, showed improvement in glucose homeostasis parameters.^4, 5^ However, these clinical studies did not show the effect of testosterone supplementation on the insulin responsiveness and gluconeogenesis in the liver, and on the serum levels of known regulators of glucose homeostasis, like insulin, glucagon, leptin, interleukin-6, and so on. To address this, we studied the effect of testosterone supplementation on insulin responsiveness and gluconeogenesis in the liver of high-fat diet-induced T2DM model in male C57BL6J mice as well as in HepG2 cell line.

## Materials and methods

### Animal experiments

Eight-week-old male C57BL6J mice were obtained from the Small Animal Facility of the National Institute of Immunology (New Delhi, India). All animals were housed and used as per the national guidelines provided by the Committee for the Purpose of Control and Supervision of Experiments on Animals. Protocols for the experiments were approved by the Institutional Animal Ethics Committee and the Committee for the Purpose of Control and Supervision of Experiments on Animals.

**Figure 1:**
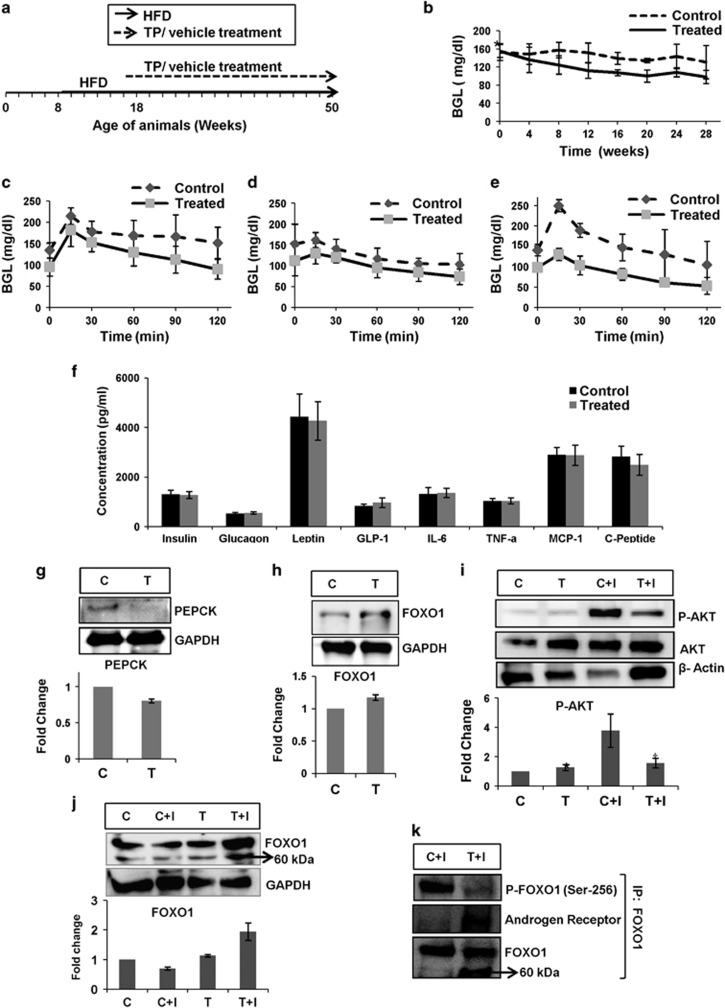
(**a**) Schematic representation of HFD feeding and treatment duration in animals. (**b**) Reduced hepatic glucose output in Treated animals. (**b**) Fasting BGL of Control and Treated animals. The data were analyzed by *t*-test, data represent mean±s.d., *n*=8, *P*<0.05; *, *P*>0.05; (**c**–**e**) PTT in Control and Treated animals after 4 (**c**), 16 (**d**) and 32 (**e**) weeks of treatment. The data were analyzed by *t*-test, data represent mean±s.d., *n*=8, *P*<0.05. (**f**) No significant change in serum level of key glucose homeostatic hormones and cytokines. Serum levels of analytes involved in glucose homeostasis in Control and Treated animals. The data were analyzed by *t*-test, data represent mean±s.d., *n*=6, *P*-value >0.1. (**g**–**j**) Immonoblot analyses depict reduced PEPCK level, but increased insulin resistance in the liver of Treated animals. (**g**) Immunoblot and densitometry for PEPCK levels in the liver of Control and Treated animals. The data were analyzed by *t*-test, data represent mean±s.d. of three independent experiments (*n*=3), *P*<0.05. (**h**) Immunoblot and densitometry for FOXO1 levels in the liver of Control and Treated animals. The data were analyzed by *t*-test, data represent mean±s.d. of three independent experiments (*n*=3), *P*<0.05. (**i**–**j**) Immunoblot and densitometry for P-AKT (Ser-473) (**i**) and FOXO1 (**j**) in the liver of Control and Treated animals upon insulin administration. The data were analyzed by two-way repeated measures ANOVA test followed by Bonferroni *post hoc* analysis; data represent mean±s.d., of three independent experiments (*n*=3), *P*<0.05; *, no significant change as compared with C; †, no significant change as compared with T. (**k**) FOXO1 and AR interaction. Liver tissue lysate of insulin-administered Control (C+I) and insulin-administered Treated (T+I) animals immunoprecipitated for FOXO1, and immunoblotted for P-FOXO1 (Ser-256), AR and FOXO1. ANOVA, analysis of variance; C, Control; C+I, Control animal with insulin treatment; HFD, high-fat diet; PTT, pyruvate tolerance test; T, Treated; T+I, Treated animal with insulin treatment.

Eight-week-old male C57BL6J mice were fed with 60% kilocalorie fat diet or high-fat diet (from Research Diets, Inc., New Brunswick, NJ, USA, Cat. No. D12492) for 10 weeks till the end of the experiment. After model confirmation by glucose tolerance test in comparison with normal chow-fed age-matched male C57Bl6J mice, animals were randomly grouped (*n*=8, owing to high mortality, that is, >20% and long duration of the experiments) into Control and Treated groups. In Treated group, 18 weeks age onwards till the end of the experiment, 8 mg kg^−1^ body weight testosterone propionate (from Sigma Aldrich, St Louis, MO, USA) suspended in sesame oil was subcutaneously injected twice a week and the Control group was treated with vehicle sesame oil (Figure 1a). All experiments were performed after 24 h of treatment. Animals were randomly selected from Treated and Control groups for experiments with blinding.

For pyruvate tolerance test, the animals were fasted for 18 h and 2 mg kg^−1^ body weight Na-pyruvate (from Sigma Aldrich) was injected intraperitoneally. Then BGLs were measured every 30 min by taking a drop of blood from the tail vein.

### Assays and analysis

Serum was isolated from animals after 28 weeks of treatment, at the age of 46 weeks, by bleeding them after 2 h of fasting, upon mild anesthesia with isofluorane. Multiplex ELISA kit for diabetes panel was procured from Millipore (Molsheim Cedex, France; Cat. No. MMHMAG-44K-14). The experiment and analysis were carried out as per the manufacturer's protocol. For tissue isolation and lysate preparation, animals were killed by cervical dislocation; after 32 weeks of treatment, at the age of 50 weeks, liver removed, snap frozen and homogenized in chilled condition. The lysate was used for immunoblot and immunoprecipitation. Animals were not fasted prior to killing. In animals given insulin treatment, 0.75 mIU insulin per  gram body weight was intraperitoneally injected and were killed after 1 h. Primary antibodies P-AKT (Ser-473; Cat. No. #4058), AKT (Cat. No. #2938), P-FOXO1 (Ser-256; Cat. No. #9461), FOXO1 (Cat. No. #2880), P-GSK3α (Ser-21; Cat. No. #9316) and GSK3α (Cat. No. #4337) were procured from Cell Signalling Technologies (Danvers, MA, USA). PEPCK (Cat. No. #PA5-15364) was procured from Thermo Fischer (Waltham, MA, USA). GAPDH (Cat. No. sc-47724), β-actin (Cat. No. sc-47778) and horse radish peroxidase-conjugated secondary antibodies were procured from Santa Cruz (Santa Cruz, CA, USA). Androgen receptor (AR) antibody (Santa cruz, Cat. No. sc-815) was a gift from Dr SS Majumdar's lab, NII.

### *In vitro* experiments

HepG2 cells (from ATCC, Manassas, VA, USA) were grown in high-glucose DMEM with 10% fetal bovine serum and 1% antibiotic antimycotic (all from GIBCO, Auckland, New Zealand) till 80% confluency. Cells were serum starved in serum-free media for 6 h before the experiment. Insulin, testosterone and LY294002 were procured from Sigma Aldrich. Cell lysates were used for immunoblot.

Cells tested negative for mycoplasma contamination (EZ-PCR mycoplasma test kit, Biological Industries, Beit-Haemek, Israel was used).

### Statistical analysis

The data have shown normal distribution. All values were presented as the mean and ±s.d. Statistical significance was estimated either by unpaired, two-tailed Student's *t*-test (for two comparisons) or two-way repeated measures analysis of variance test (for more than two comparisons) followed by Bonferroni *post hoc* analysis. *P*-values <0.05 were considered significant.

## Results and discussion

### Decreased hepatic glucose output in Treated animals as compared with Control

Fasting BGL is a marker of hepatic glucose output.^2, 3^ The testosterone-Treated animals showed lower fasting BGL as compared with the Control for over a period of 28 weeks (Figure 1b). Hepatic glucose output can be quantified by the pyruvate tolerance test, where, upon fasting, pyruvate is administered to animals. This pyruvate is converted to glucose in the liver and BGL rises. The Treated animals showed lesser increase in BGL as compared with Control in pyruvate tolerance test (Figures 1c–e). Lower fasting BGL and reduced increase in BGL upon pyruvate administration in the Treated group compared with the Control indicate reduced hepatic glucose output during long hours of fasting, and hence better glycemic control upon testosterone administration in T2DM male mice.

### No significant change in levels of key glucose homeostatic hormones in the Treated and Control animals

Body glucose homeostasis is regulated by various hormones, and cytokines like insulin, glucagon, leptin, interleukin-6, TNFα, and so on. Hyperglycemia induces the β-islets of pancreas to produce insulin and to decrease BGL, and hypoglycemia induces the α-islets of pancreas to secrete glucagon, which helps liver to release glucose to attain normoglycemia.^6^ Leptin, an adipokine, acts on the feeding center of the brain, hypothalamus and prevents hyperphagia.^7, 8^ Interleukin-6, TNFα and MCP-1 are pro-inflammatory cytokines, which increase hepatic insulin resistance and increase hepatic glucose output.^9, 10^ C-peptide, the C-terminal peptide of the proinsulin molecule that is cleaved off to form insulin, is a measure for insulin secretion.^11^ GLP-1 (glucagon-like peptide-1), an incretin molecule, increases insulin secretion and reduces glucagon secretion by the pancreas.^12^ As body glucose homeostasis is controlled by these hormones and cytokines, we investigated if testosterone administration had any significant effect on the serum levels of these analytes.

Interestingly, we observed no significant change in the levels of these analytes in the serum of Treated and Control animals (Figure 1f). This suggests that testosterone supplementation did not affect hepatic gluconeogenesis through these glucose homeostatic hormones. Instead, it could have altered signaling in the liver, which led to reduced hepatic glucose output in the testosterone-administered T2DM males. Thus, we investigated the effect of testosterone on gluconeogenesis pathway and insulin responsiveness in the liver.

### Immunoblot analysis revealed reduced PEPCK level, but increased insulin resistance in the liver of Treated animals as compared with Control ones

In normal subjects, PEPCK level rises during fasting periods to attain normoglycemia, and in increased insulin resistance conditions also, its level increases resulting in increased hepatic glucose output. Lin *et al.*^13^ showed that AR knockout in the liver of male C57BL6J mice resulted in hepatic steatosis, and loss of AR led to increased PEPCK level and hepatic insulin resistance. Thus, we checked the PEPCK level in the liver of Treated and the Control animals, and found significantly lower level of PEPCK in the liver of the Treated animals (Figure 1g). Thus, significant decrease in PEPCK might have resulted in reduced hepatic glucose output in the Treated animals, as depicted by fasting BGL and pyruvate tolerance test.

The transcription factor FOXO1 positively regulates PEPCK. FOXO1 is downstream of AKT in the insulin-signaling pathway and is inhibited by phosphorylation at Ser-256 by AKT, resulting in its nuclear exclusion.^14^ As FOXO1 is a regulator of PEPCK, we checked FOXO1 level upon testosterone administration. Surprisingly, we found an increased level of total FOXO1 in the liver of Treated animals as compared with the Control (Figure 1h).

Increased level of FOXO1 is related to insulin resistance. Insulin resistance leads to decreased P-AKT (Ser-473) levels or causes AKT inactivation, and hence the inactivation of FOXO1 is reduced. In increased insulin resistance condition, there is reduction in P-AKT levels upon insulin treatment and increased FOXO1.^15^ Thus, we investigated the P-AKT (Ser-473) level, which is also a marker for tissue insulin responsiveness in the liver of Treated and Control animals.

We gave exogenous insulin treatment to the animals, and then checked for P-AKT (Ser-473) and FOXO1 levels in the liver of the two groups. We observed that the P-AKT levels were highest and FOXO1 levels were least in the Control animals that were also given insulin. The Treated animals upon insulin administration had the highest FOXO1 and significantly lower P-AKT (Ser-473) level than the insulin-treated Control (Figures 1i–j). There was no significant difference in the P-AKT (Ser-473) level of Control and Treated animals. The increase in P-AKT (Ser-473) level in Treated animals upon insulin administration was insignificant, which indicates impaired insulin action/signaling in the liver of Treated animals, whereas a significant increase in P-AKT (Ser-473) level upon insulin administration in Control animals suggest better insulin response in the liver (Figure 1i). The PEPCK levels were beyond detection range in insulin-administered Control and Treated animals.

To detect the P-FOXO1 (Ser-256) levels in the tissue lysates, we immunoprecipitated tissue lysates of insulin-administered Control (C+I) and Treated (T+I) animals for FOXO1. We probed for P-FOXO1 (Ser-256) and found significantly higher level of P-FOXO1 (Ser-256) in the insulin-administered Control as compared with the insulin-administered Treated animals (Figure 1k). As PI3K-AKT pathway regulates P-FOXO1 (Ser-256) level, our previous finding of increased insulin resistance in the liver of Treated animals is further validated.

Li *et al.* and Huang *et al.*^16, 17^ demonstrated interaction between FOXO1 and AR in prostate cancer cells. Li *et al.*^16^ reported that interaction and binding of AR to FOXO1 inhibited the ability of FOXO1 to bind to target DNA sequence and hence reduced transcriptional activity of FOXO1. Huang *et al.*^17^ reported that the interaction between FOXO1 and AR led to proteasomal degradation of FOXO1 to a 60 kDa product, and transcriptional activity of FOXO1 was inhibited. They also reported that this interaction between FOXO1 and AR was independent of PI3K-AKT signaling, and the phosphorylation status of FOXO1 had no role in this interaction. When we immunoblotted for FOXO1 in Control and Treated animals with/without insulin administration, we found a profound 60 kDa band in the T+I sample (Figure 1j), which is possibly owing to proteasomal degradation of FOXO1 upon interaction with AR. To validate if there was any interaction between FOXO1 and AR in the liver of Treated and Control animals, the immunoprecipitated samples for FOXO1 were probed for AR. The T+I sample immunoprecipitated for FOXO1 was positive for AR (Figure 1k), which indicates that there was significant interaction between FOXO1 and AR in the hepatic tissue of the Treated animals. The immunoblot for FOXO1 of the immunoprecipitated samples also depicted a profound 60 kDa band in the T+I sample, which was very faint in the C+I sample and indicate proteasomal degradation of FOXO1 owing to interaction with AR (Figure 1k). The AR–FOXO1 interaction prevented DNA binding of FOXO1 and had an inhibitory effect on transcriptional activity of FOXO1,^16^ which could possibly be the reason for lower PEPCK levels in the liver of Treated animals, despite the increase in insulin resistance.

To confirm the above results, we performed experiments in HepG2 cell line.

### HepG2 cell line study revealed increase in hepatic insulin resistance owing to testosterone treatment

**Figure 2:**
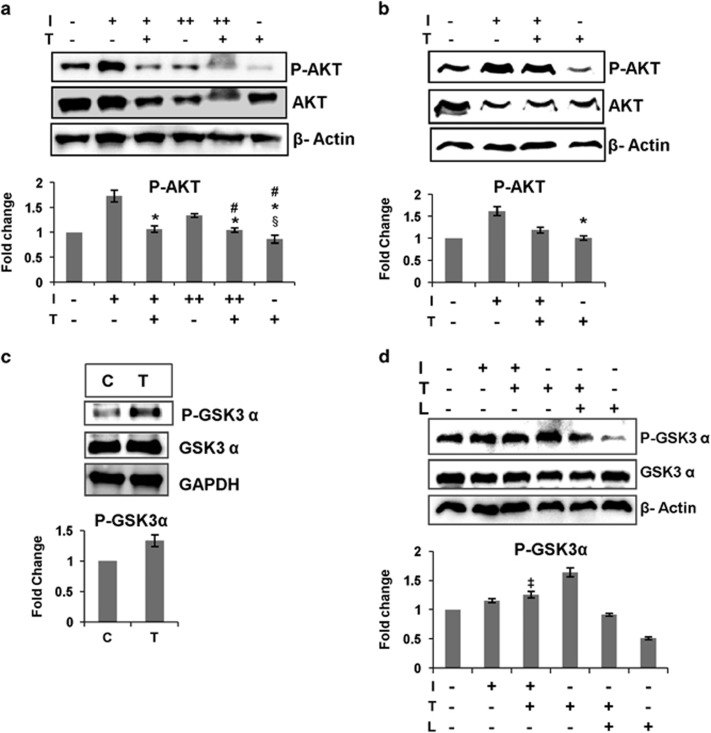
(**a**–**b**) Testosterone increases insulin resistance in HepG2 cells. (**a**) Immunoblot and densitometry for P-AKT (Ser-473) in HepG2 cells, I(+)=insulin (10 ng ml^−1^), I(++)=insulin (250 ng ml^−1^), T(+)=testosterone (50 ng ml^−1^). Testosterone treatment to cells for 120 min, and insulin treatment 10 ng ml^−1^ and 250 ng ml^−1^ for 60 min, without removing testosterone; the data were analyzed by two-way repeated measures ANOVA test followed by Bonferroni *post hoc* analysis; data represent mean±s.d. of three independent experiments (*n*=3), *P*<0.05; *, no significant change as compared with I(−)T(−); #, no significant change as compared with I(+)T(+); §, no significant change as compared with I(++)T(+). (**b**) Immunoblot and densitometry for P-AKT (Ser-473) in HepG2 cells, I(+)=insulin (10 ng ml^−1^), T(+)=testosterone (50 ng ml^−1^). Testosterone pretreatment was given for 60 min and insulin treatment for 30 min, without removing testosterone; the data were analyzed by two-way repeated measures ANOVA test followed by Bonferroni *post hoc* analysis; data represent mean±s.d. of three independent experiments (*n*=3), *P*<0.05; *, no significant change as compared with I(−)T(−); #, no significant change as compared with I(+)T(+); (**c**–**d**) testosterone increases hepatic GSK3α inhibition. (**c**) Immunoblot and densitometry for P-GSK3α (Ser-21) in the liver of Control (C) and Treated (T) animals. The data were analyzed by *t*-test, data represent mean±s.d. of three independent experiments (*n*=3), *P*<0.05. (**d**) Immunoblot and densitometry for P-GSK3α (Ser-21) in HepG2 cells, I(+)=insulin (10 ng ml^−1^), T(+)=testosterone (50 ng ml^−1^), L=10 μm LY294002, LY294002 added 15 min prior to testosterone treatment. Testosterone treatment was given for 60 min and insulin treatment for 30 min without removing testosterone; the data were analyzed by two-way repeated measures ANOVA test followed by Bonferroni *post hoc* analysis; data represent mean±s.d. of three independent experiments (*n*=3), *P*<0.05; ‡, no significant change as compared with I(+).

The animal studies showed increased hepatic insulin resistance in the Treated animals. To further validate this finding, we created insulin resistance condition in HepG2 cells by giving prolonged insulin treatment (for 60 min) at low (10 ng ml^−1^), I(+), or high (250 ng ml^−1^) concentration, I(++), to testosterone-pretreated cells, T(+) (testosterone pretreatment for 60 min and thereafter testosterone was not removed). We found that activation of AKT or P-AKT (Ser-473) levels was significantly reduced in the I(+)T(+) and I(++)T(+) samples as compared with I(+) and I(++), respectively (Figure 2a). There was no significant difference in P-AKT (Ser-473) levels in I(−)T(−), I(+)T(+), I(++)T(+) and T(+) samples.

We also studied the effect of testosterone on cells without inducing insulin resistance by insulin treatment at low concentration (10 ng ml^−1^), I(+), for 30 min in testosterone-pretreated cell, T(+), (pretreatment for 60 min and thereafter testosterone was not removed), and found that testosterone increased insulin resistance, depicted by significantly lower P-AKT (Ser-473) levels in I(+)T(+) as compared with I(+) (Figure 2b). The presence of testosterone significantly reduced insulin-induced AKT activation. Testosterone had negatively affected insulin-induced AKT activation, but there was significant difference in P-AKT (Ser-473) levels of I(−)T(−) and I(+)T(+) samples, possibly because prolonged insulin treatment was not given to induce insulin resistance. The P-AKT (Ser-473) level of T(+) was not significantly different from that of I(−)T(−). Thus, all the above experiments suggest that testosterone is increasing hepatic insulin resistance.

Next, we went on to check the level of P-GSK3α (Ser-21) in the liver of Treated and Control animals as glycogen synthase kinase-3 (GSK3) also has an important role in PEPCK regulation and glucose homeostasis in the liver.

### Testosterone treatment increased hepatic GSK3α inactivation, and GSK3α inactivation is partially dependent on PI3K/AKT pathway

GSK3, a serine-threonine kinase, not only has an inhibitory role in the glycogen synthesis pathway by inhibiting glycogen synthase, the key enzyme in glycogen synthesis, and also negatively regulates key signaling molecules in the cell like AKT, mTOR and insulin receptor substrate 1. One of the major regulators of GSK3 is AKT, which phosphorylates and inactivates GSK3. Increased insulin resistance perturbs the PI3K-AKT pathway and increases activation of GSK3, which further disrupts glucose homeostasis by negatively regulating key insulin-signaling pathway molecules.^15, 18, 19^

In our experiment, we found increased GSK3α inactivation, that is, higher levels of P-GSK3α (Ser-21) in the liver of Treated animals (Figure 2c). Though our previous experiments depict increased insulin resistance in the liver of Treated animals, the testosterone treatment resulted in increased GSK3α inactivation. So, we checked the role of PI3K/AKT pathway in testosterone-mediated GSK3α inactivation by either stimulating the cells with insulin or inhibiting PI3K with LY294002 in HepG2 cell line, as mentioned. Insulin alone stimulated GSK3α inactivation as in I(+) so as insulin with testosterone, I(+)T(+), but there was no significant difference in GSK3α inactivation between these two groups, whereas T(+) showed highest GSK3α inactivation. GSK3α inactivation was significantly more in T(+)L(+) group as compared with L(+) one. The P-GSK3α (Ser-21) levels were highest when cells were treated with only testosterone and testosterone could inactivate GSK3α even when the PI3K/AKT pathway was blocked (Figure 2d), suggesting testosterone-mediated inhibition of GSK3α in the liver was independent of PI3K/AKT pathway.

Previous studies have shown the inhibitory effect of GSK3 inactivation on hepatic gluconeogenesis. Bosch *et al.*^20^ have shown that GSK3 inactivation by intraperitoneal injection of LiCl to rats downregulated hepatic PEPCK level and reduced hepatic glucose output.^20^ Lochhead *et al.*^21^ further investigated the regulatory role of GSK3 in the expression of PEPCK, and reported that inactivation of GSK3 by LiCl and specific inhibitors (SB-216763 or SB-415286) resulted in decreased PEPCK gene expression in H4IIE rat hepatoma cells, which reduced hepatic gluconeogenesis. They also reported that the GSK3 inactivation had no effect on AKT activation.^21^ These studies demonstrate that inactivation of GSK3 leads to reduced hepatic gluconeogenesis. Thus, in our study, we infer that despite increased hepatic insulin resistance, PI3K-AKT-independent inhibition of GSK3α has reduced PEPCK level, and hence, hepatic gluconeogenesis in the liver of Treated animals.

Our previous experiments showed that increased hepatic insulin resistance increased FOXO1 level, but this did not lead to increased PEPCK gene expression owing to interaction of AR to FOXO1 in the Treated animals. The increased hepatic insulin resistance was counterbalanced by PI3K-AKT-independent GSK3 inhibition and AR-mediated inhibition of FOXO1, reducing hepatic PEPCK level and hepatic gluconeogenesis. In conclusion, in liver, a dual role of testosterone is observed. On the one hand, testosterone increased insulin resistance in the liver and on the other hand interacted with FOXO1, inhibiting its transcriptional activity and inactivated GSK3α, downregulating PEPCK and reducing gluconeogenesis, which is otherwise upregulated in T2DM. The AR-mediated FOXO1 inhibition and PI3K-AKT-independent inhibition of GSK3α have downregulated PEPCK, and reduced hepatic glucose output and enhanced glucose homeostasis in the liver. Thus, the increased insulin resistance in liver owing to testosterone treatment does not worsen T2DM condition, instead improves glucose homeostasis in the Treated animals.
